# Electrocardiographic parameter profiles for differentiating hypertrophic cardiomyopathy stages

**DOI:** 10.1002/joa3.70031

**Published:** 2025-03-04

**Authors:** Naomi Hirota, Shinya Suzuki, Takuto Arita, Naoharu Yagi, Mikio Kishi, Hiroaki Semba, Hiroto Kano, Shunsuke Matsuno, Yuko Kato, Takayuki Otsuka, Junji Yajima, Tokuhisa Uejima, Yuji Oikawa, Takeshi Yamashita

**Affiliations:** ^1^ Department of Cardiovascular Medicine The Cardiovascular Institute Tokyo Japan

**Keywords:** diagnostic modeling, dilated phase hypertrophic cardiomyopathy, disease progression, electrocardiogram parameters, hypertrophic cardiomyopathy

## Abstract

**Background:**

The efficacy of artificial intelligence (AI)‐enhanced electrocardiography (ECG) for detecting hypertrophic cardiomyopathy (HCM) and its dilated phase (dHCM) has been developed, though specific ECG characteristics associated with these conditions remain insufficiently characterized.

**Methods:**

This retrospective study included 19,170 patients, with 140 HCM or dHCM cases, from the Shinken Database (2010–2017). The 140 cases (HCM‐total) were categorized into basal‐only HCM (HCM‐basal, *n* = 75), apical involvement (HCM‐apical, *n* = 46), and dHCM (*n* = 19). We analyzed 438 ECG parameters across the P‐wave (110), QRS complex (194), and ST‐T segment (134). High parameter importance (HPI) was defined as 1/*p* > 10^4^ in univariate logistic regression, while multivariate logistic regression was used to determine the area under the receiver operating characteristic curves (AUROC).

**Results:**

In HCM‐basal and HCM‐apical, HPI was predominantly observed in the ST‐T segment (49% and 51%, respectively), followed by the QRS complex (29% and 27%). For dHCM, HPI was lower in the ST‐T segment (16%) and QRS complex (22%). The P‐wave had low HPI across all subtypes. AUROCs for models with total ECG parameters were 0.925 for HCM‐basal, 0.981 for HCM‐apical, and 0.969 for dHCM. While AUROCs for the top 10 HPI models were lower than the total ECG parameter model for HCM total, they were comparable across HCM subtypes.

**Conclusions:**

As HCM progresses to dHCM, a shift in HPI from the ST‐T segment to the QRS complex provides clinically relevant insights. For HCM subtypes, the top 10 ECG parameters yield predictive performance similar to the full parameter set, supporting efficient approaches for AI‐based diagnostic models.

## Graphical abstract

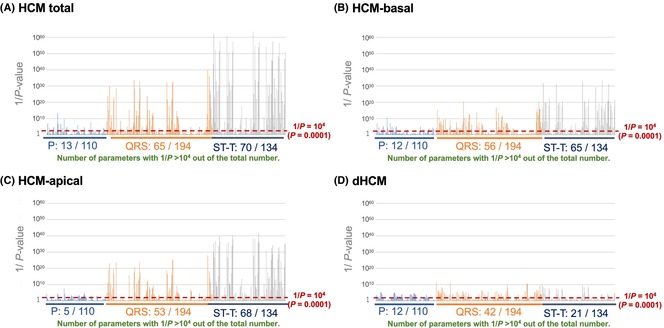

## 1 INTRODUCTION

Hypertrophic cardiomyopathy (HCM) is one of the most common genetic heart diseases. Although most patients with HCM are asymptomatic, it is one of the leading causes of sudden death among adolescents and young adults^1^ and is associated with considerable morbidity across all age groups.^2^ Additionally, HCM carries the risk of heart failure, atrial fibrillation, and stroke.^3^, ^4^ Early detection and long‐term follow‐up of patients with HCM are crucial, and strategies such as screening at‐risk family members, pharmacological therapy, and assessing and mitigating the risk of sudden death are important.^3^, ^5^, ^6^

Furthermore, a lifelong process of left ventricular remodeling and progressive dysfunction can occur in a minority of HCM patients.^7^, ^8^, ^9^ This stage of HCM, known as dilated phase HCM (dHCM), has been reported to be associated with a poor prognosis.^10^, ^11^ Because of the slowly evolving nature of HCM, timely identification of patients at risk of left ventricular dysfunction or heart failure might improve their poor prognosis.^12^

Although echocardiography and cardiac magnetic resonance imaging are useful for diagnosing dHCM, these tests can be costly for annual check‐ups of all HCM patients. Moreover, some patients, especially those who are asymptomatic, may discontinue annual follow‐ups with cardiac specialists over time. Therefore, there is a need to develop readily available and cost‐effective screening methods for HCM and dHCM that are accessible even in non‐specialized cardiac clinics.

The electrocardiogram (ECG), a non‐invasive and widely available test, is commonly used for detecting and managing cardiac diseases. Notably, over 90% of HCM patients exhibit abnormalities on ECG.^13^ Traditionally, ECG screening for HCM has relied on the manual or automated detection of specific features, such as criteria for left ventricular hypertrophy (LVH), left axis deviation, prominent Q waves, and T wave inversions.^14^, ^15^, ^16^

Artificial‐intelligence (AI) technology may play a significant role in linking ECG findings to clinical diagnoses. Convolutional neural network (CNN) models using ECG to detect HCM have been developed.^17^, ^18^ Moreover, we have recently developed an AI‐enhanced ECG to detect HCM and dHCM.^19^, ^20^ These models have shown high diagnostic performance, with AUCs around 0.9. While AI technology may be useful in detecting HCM and dHCM, it is important to understand the specific ECG features that AI focuses on in patients' ECGs.

The purpose of this study is to explore the specific ECG parameter characteristics of HCM and dHCM using traditional statistical methods, which would be the basis for understanding the AI diagnosis for these diseases.

## 2 METHODS

### 2.1 Ethics and informed consent

This study was performed in accordance with the Declaration of Helsinki (revised in 2013) and the Ethical Guidelines for Medical and Health Research Involving Human Subjects (Public Notice of the Ministry of Education, Culture, Sports, Science and Technology, and the Ministry of Health, Labour and Welfare, Japan, issued in 2017). Written informed consent was obtained from all participants. The study protocol was reviewed by the Institutional Review Board of the Cardiovascular Institute.

### 2.2 Study population

The Shinken database includes all patients who newly visited the Cardiovascular Institute, Tokyo, Japan, except for foreign travelers and patients with active cancer. This single‐hospital database was established in June 2004, and details of this database have been described elsewhere.^21^ In the present study, data from 19,170 subjects registered between February 2010 and March 2018 were extracted from the Shinken database because a computerized electrocardiogram database has been available since February 2010.

### 2.3 Diagnosis of HCM and dHCM

The diagnostic definition of HCM included (1) an interventricular septal thickness (IVST) ≥15 mm and without other causes of LVH such as moderate or severe aortic valve stenosis, uncontrolled hypertension, controlled hypertension with improved LVH, cardiac amyloidosis, or Fabry disease; (2) an IVST ≥13 mm and a family history of HCM; and (3) hypertrophy in the apex of the left ventricle.

We reviewed the clinical data of all patients with IVST ≥15 mm, not only at the initial visit but also over several years of follow‐up, to carefully determine the underlying cause of LVH in each case. Generally, we did not diagnose HCM in LVH cases with moderate or severe AS, as most were undergoing their first assessment for LVH. However, there were a few cases already diagnosed with HCM at a younger age that later progressed to AS in older age. In such cases, we included them in the HCM group if they had moderate AS at the initial visit, which would not independently explain LVH with IVST ≥15 mm. LVH patients with IVST ≥15 mm and a history of hypertension were primarily diagnosed as hypertensive heart disease. However, over several years of follow‐up, there were cases in which IVST remained elevated or worsened (IVST ≥15 mm) despite well‐controlled blood pressure (<130 mmHg). Such cases were included in the HCM group if no other causes for LVH were identified.

HCM patients were further divided into two patterns according to the location of ventricular hypertrophy^1^: basal hypertrophy only (HCM‐basal) and^2^ hypertrophy involving the apex (HCM‐apical), with the latter defined by the apex thickness exceeding the IVST measurement. Patients with dHCM were also included in this study, defined as previously diagnosed HCM patients with advancement of a left ventricular ejection fraction (LVEF) <50% or the initially diagnosed hypertrophy with decreased LVEF who were excluded from other causes of LVH using echocardiography, cardiac MRI, or biopsy.

### 2.4 ECG sampling

Standard 12‐lead ECGs were recorded over 10 s with patients in a supine position using an ECG machine (CardioSoft V6.71 and MAC 5500 HD; GE Healthcare, Chicago, IL, USA) at a sampling rate of 500 Hz. Raw digital records were stored in the MUSE data management system.

### 2.5 ECG parameters

The GE system's ECG database included 639 automatically measured parameters. Of these, we excluded 201 parameters (9 non‐lead‐specific and 192 lead‐specific) that were relative coordinate points (i.e., P‐wave start point). This left 438 parameters (6 non‐lead‐specific and 432 lead‐specific) for analysis (Figure S1).

### 2.6 Evaluation and statistical analysis

Statistical analyses were performed using SPSS version 26.0 (IBM, Chicago, IL), with a *p*‐value <.05 considered statistically significant. Categorical and continuous data are presented as numbers (%) and means ± SD, respectively.

#### 2.6.1 Analysis 1

We developed predictive models for four diagnostic labels: HCM‐total, HCM‐basal, HCM‐apical, and dHCM, using univariate logistic regression analysis with each of the 438 ECG parameters. *P*‐values for each model were presented separately for the P‐wave, QRS complex, and ST‐T segment for each disease label.

#### 2.6.2 Analysis 2

For each diagnostic label, we developed multivariate logistic regression models, including four variants: (1) using a total 438 parameters, and (2–4) using subsets of 110 P‐wave, 194 QRS complex, and 134 ST‐T segment parameters. To address collinearity, we first calculated Spearman's correlation coefficient for all parameter pairs (191,406 combinations) and identified pairs with coefficients ≥0.9 as collinear. For each pair, we retained the parameter with the higher Wald statistic from univariate analysis (Analysis 1) and excluded the other. Multivariate logistic regression was then conducted using stepwise selection, including both collinear parameters retained from the initial step and parameters without collinear counterparts. Receiver operating characteristic (ROC) curves and area under the curve (AUC) values were reported for each model.

#### 2.6.3 Analysis 3

The top 10 parameters for each diagnostic label were selected based on Wald statistics from the multivariate models. Using these, we developed additional multivariate models using the top 5 and top 10 parameters. AUCs derived from ROC curves were presented for models using total ECG parameters, the top 10 parameters, and the top 5 parameters.

#### 2.6.4 Analysis 4

The same process of Analysis 1 and Analysis 2 was applied to patients without atrial tachyarrhythmia or cardiac pacing at the timing of ECG recording. The definition of atrial tachyarrhythmia was atrial fibrillation, atrial flutter, and atrial tachycardia.

## 3 RESULTS

### 3.1 Patient characteristics

The patient characteristics of the study population are summarized in Table 1. Among the 19,170 subjects included, 121 were classified into the HCM group (75 with HCM‐basal and 46 with HCM‐apical), while 19 were classified into the dHCM group. Patients in the HCM group showed greater left ventricular wall thickness. By contrast, dHCM patients had enlarged left ventricles and left atria, reduced LVEF, and a higher prevalence of aortic, mitral, or tricuspid regurgitation, as well as chronic kidney disease.

**TABLE 1:** Patient characteristics.

	Non‐HCM/dHCM, *N* = 19,030	HCM‐basal, *N* = 75	HCM‐apical, *N* = 46	dHCM, *N* = 19
Age, years	58.2 ± 15.7	61.1 ± 13.9	63.7 ± 14.0	63.6 ± 8.1
Male, *n* (%)	11,374 (59.8)	49 (65.3)	31 (67.4)	17 (89.5)
Height, cm	163.7 ± 9.4	162.8 ± 10.0	162.3 ± 9.5	166.3 ± 8.1
Weight, kg	62.6 ± 13.7	64.7 ± 13.5	66.0 ± 13.8	66.5 ± 14.3
BMI, kg/m^2^	23.3 ± 3.9	24.3 ± 3.8	24.9 ± 3.9	24.0 ± 4.4
Systolic BP, mmHg	127.1 ± 21.0	129.7 ± 20.5	136.2 ± 23.0	131.3 ± 25.1
Diastolic BP, mmHg	75.3 ± 16.0	78.2 ± 14.0	78.8 ± 13.6	77.5 ± 16.7
IVST, mm	9.6 ± 2.1	17.6 ± 3.0	17.0 ± 1.9	11.5 ± 2.3
PWT, mm	9.0 ± 1.5	11.6 ± 2.5	11.9 ± 2.5	10.6 ± 1.7
LVDd, mm	46.1 ± 5.8	42.4 ± 5.2	43.1 ± 4.7	61.0 ± 9.0
LVDs, mm	29.5 ± 6.5	24.6 ± 5.0	25.1 ± 4.2	50.6 ± 10.6
LVEF, %	65.4 ± 10.4	72.9 ± 7.7	72.1 ± 8.0	31.9 ± 10.5
LAD, mm	35.4 ± 7.1	41.2 ± 6.9	43.8 ± 8.0	49.9 ± 11.2
eGFR, mL/min/1.73 m^2^	70.8 (19.8)	71.7 (17.0)	60.8 (17.3)	55.8 (17.0)
Congestive heart failure, *n* (%) (heart failure admission within 90 days)	436 (2.2)	1 (1.3)	1 (2.2)	3 (15.8)
Heart failure with reduced EF, *n* (%)	1101 (5.8)	1 (1.3)	0 (0.0)	18 (94.7)
Ischemic heart disease, *n* (%) (PCI within 90 days)	1844 (9.7)	5 (6.7)	3 (6.5)	2 (10.5)
Asymptomatic ischemia, *n* (%)	419 (2.2)	1 (1.3)	1 (2.2)	1 (5.3)
Old myocardial infarction, *n* (%)	452 (2.4)	0 (0.0)	0 (0.0)	0 (0.0)
Acute coronary syndrome, *n* (%)	618 (3.2)	0 (0.0)	0 (0.0)	0 (0.0)
Aortic stenosis, *n* (%)	627 (3.3)	2 (2.7)	1 (2.2)	1 (5.3)
Aortic regurgitation, *n* (%)	419 (3.3)	2 (2.7)	0 (0.0)	2 (10.5)
Mitral regurgitation, *n* (%)	549 (2.9)	6 (8.0)	5 (10.9)	3 (15.8)
Mitral stenosis, *n* (%)	82 (0.4)	0 (0.0)	0 (0.0)	0 (0.0)
Tricuspid regurgitation, *n* (%)	428 (2.2)	0 (0.0)	1 (2.2)	11 (57.9)
Dilated cardiomyopathy, *n* (%)	151 (0.8)	0 (0.0)	0 (0.0)	0 (0.0)
Hypertensive cardiomyopathy, *n* (%)	2208 (11.6)	0 (0.0)	0 (0.0)	0 (0.0)
Ischemic cardiomyopathy, *n* (%)	240 (1.3)	0 (0.0)	0 (0.0)	0 (0.0)
Aortic aneurism, *n* (%)	289 (1.5)	0 (0.0)	0 (0.0)	0 (0.0)
Aortic dissection, *n* (%)	191 (1.0)	0 (0.0)	0 (0.0)	0 (0.0)
Hypertension, *n* (%)	8418 (44.2)	39 (52.0)	28 (60.9)	12 (63.2)
Diabetes, *n* (%)	2359 (12.4)	12 (16.0)	7 (15.2)	3 (15.8)
Smoking history, *n* (%)	7975 (41.9)	34 (45.3)	21 (45.7)	15 (78.9)
Chronic kidney disease, *n* (%)	3296 (17.3)	13 (17.3)	17 (37.0)	13 (68.4)
Paroxysmal AF, *n* (%)	1780 (9.4)	3 (4.0)	6 (13.0)	2 (10.5)
Persistent AF, *n* (%)	1017 (5.3)	4 (5.3)	6 (13.0)	5 (26.3)
Atrial tachyarrhythmia at the timing of ECG recording, *n* (%)	1591 (8.4)	5 (6.7)	13 (28.3)	7 (36.8)
Cardiac pacing at the timing of ECG recording, *n* (%)	102 (0.5)	1 (1.3)	1 (2.2)	2 (10.5)

*Note*: Data are presented as the mean ± standard deviation unless otherwise stated.

Abbreviations: AF, atrial fibrillation.; BMI, body mass index; BP, blood pressure; dHCM, dilated phase HCM; EF, ejection fraction; HCM, hypertrophic cardiomyopathy; HCM‐apical, HCM with hypertrophy involving the apex; HCM‐basal, HCM with basal hypertrophy only; IVST, intraventricular septum thickness; LAD, left atrial diameter; LVDd, left ventricular end‐diastolic diameter; LVDs, left ventricular end‐systolic diameter; LVEF, left ventricular ejection fraction; PCI, percutaneous coronary intervention; PWT, posterior left ventricular wall thickness.

### 3.2 Analysis 1: Importance of ECG parameters for predicting HCM and dHCM

**FIGURE 1:**
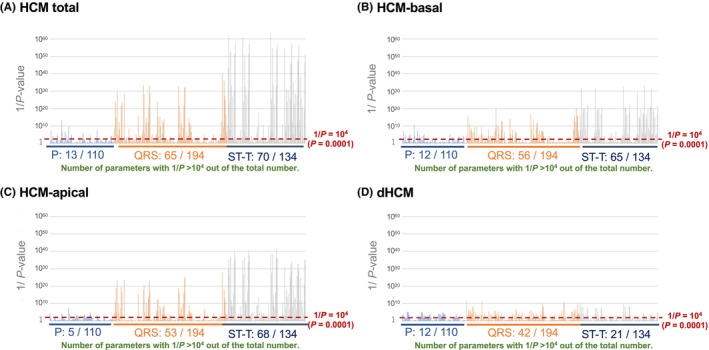
Distribution of ECG parameter importance. (A) HCM total, (B) HCM‐basal, (C) HCM‐apex, and (D) dHCM. Parameter importance is represented as the reciprocal of the *p*‐value (1/*p*) derived from univariate models using each ECG parameter. 1/*p*, reciprocal *p*‐value; dHCM, dilated phase hypertrophic cardiomyopathy; HCM, hypertrophic cardiomyopathy; HCM‐apical, HCM with hypertrophy involving the apex; HCM‐basal, HCM with basal hypertrophy only; P, P‐wave; QRS, QRS complex; ST‐T, ST‐T segment.

The reciprocal *P*‐values for each of the 438 ECG parameters, derived from univariate logistic regression analysis for HCM‐total, HCM‐basal, HCM‐apical, and dHCM, are presented in Figure 1 as exponential minus 10 values, categorized by ECG phases (P‐wave, QRS complex, and ST‐T segment). Higher parameter importance (HPI) was defined as a reciprocal *p*‐value ≥10^4^ (*p*‐value <.0001; Wald statistics >15.13) for each disease label.

In HCM‐total, the proportions of ECG parameters with HPI were 11.2% for the P‐wave (13/110), 33.5% for the QRS complex (65/194), and 52.2% for the ST‐T segment (70/134). For HCM‐basal, these proportions were 10.9% for the P‐wave (12/110), 28.9% for the QRS complex (56/194), and 48.5% for the ST‐T segment (65/134). For HCM‐apical, the proportions were 4.5% for the P‐wave (5/110), 27.3% for the QRS complex (53/194), and 50.7% for the ST‐T segment (68/134). For dHCM, the proportions were 10.9% for the P‐wave (12/110), 21.6% for the QRS complex (42/194), and 15.7% for the ST‐T segment (21/134).

### 3.3 Analysis 2: Predictive performance of models using ECG parameters for HCM and dHCM

**FIGURE 2:**
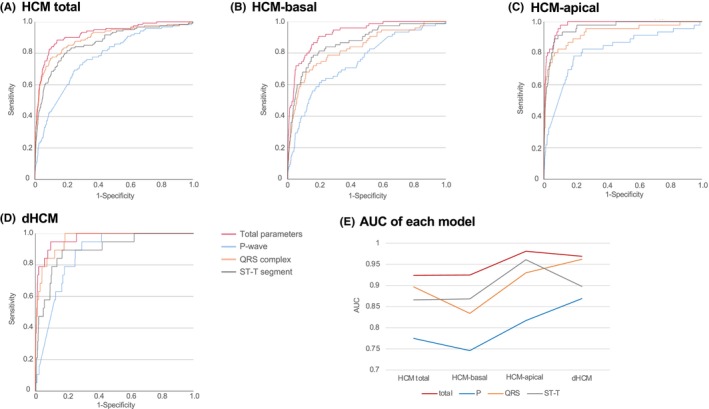
ROC curves of prediction models for each distinct disease label (A: HCM total, B: HCM‐basal, C: HCM‐apex, and D: dHCM) and AUCs for each model (E), using total parameters as well as parameters specific to the P‐wave, QRS complex, and ST‐T segment. 1/*p*, reciprocal *p*‐value; AUC, area under the curve; dHCM, dilated phase hypertrophic cardiomyopathy; HCM, hypertrophic cardiomyopathy; HCM‐apical, HCM with hypertrophy involving the apex; HCM‐basal, HCM with basal hypertrophy only; P, P‐wave; QRS, QRS complex; ROC, receiver operating characteristic; ST‐T, ST‐T segment.

Out of the ECG parameters, 281 non‐collinear parameters were selected for HCM‐total, 285 for HCM‐basal, 284 for HCM‐apical, and 280 for dHCM. These were used to develop multivariate models for each disease label. ROC curves for each multivariate model are shown in Figure 2, with models developed for total ECG parameters and subsets from the P‐wave, QRS complex, or ST‐T segment.

For HCM‐total, the AUCs were 0.924 (95%CI, 0.898–0.950) for the total model, 0.775 (95%CI, 0.733–0.818) for the P‐wave model, 0.897 (95%CI, 0.863–0.931) for the QRS complex model, and 0.866 (95%CI, 0.828–0.903) for the ST‐T segment model. For HCM‐basal, the AUCs were 0.925 (95%CI, 0.898–0.953) for the total model, 0.746 (95%CI, 0.686–0.805) for the P‐wave model, 0.834 (95%CI, 0.782–0.886) for the QRS complex model, and 0.868 (95%CI, 0.824–0.913) for the ST‐T segment model. For HCM‐apical, the AUCs were 0.981 (95%CI, 0.971–0.990) for the total model, 0.817 (95%CI, 0.744–0.889) for the P‐wave model, 0.930 (95%CI, 0.884–0.976) for the QRS complex model, and 0.961 (95%CI, 0.939–0.983) for the ST‐T segment model. For dHCM, the AUCs were 0.969 (95%CI, 0.942–0.996) for the total model, 0.869 (95%CI, 0.821–0.918) for the P‐wave model, 0.962 (95%CI, 0.935–0.988) for the QRS complex model, and 0.898 (95%CI, 0.828–0.968) for the ST‐T segment model.

### 3.4 Analysis 3: Identification of the top 10 important parameters

Table 2 presents the top 10 most important parameters for each disease label in the multivariate models using total parameters. Representative ECG waveforms for each disease label are shown in Figure S2A–D.

**TABLE 2:** Top 10 important parameters for the diagnosis.

ECG segment	Parameter	Wald statistics	Odds ratio
HCM‐total			
P	P area in I	18.056	1.101 (1.053–1.152)
	P area (Full) in aVL	17.177	0.911 (0.871–0.952)
	P area (Full) in II	16.415	0.956 (0.935–0.977)
QRS	Q area in V6	51.360	1.005 (1.004–1.007)
	Max S amplitude in V4	44.075	1.002 (1.001–1.002)
	R axis	40.760	0.972 (0.963–0.980)
	QRS area in V3	35.086	1.001 (1.000–1.001)
	Q area in III	28.114	0.998 (0.997–0.999)
	S area in V5	26.469	1.001 (1.001–1.002)
	Max R amplitude in II	25.722	1.001 (1.001–1.002)
HCM‐basal			
P	P area in I	17.159	1.004 (1.002–1.006)
QRS	R peak time in V4	35.355	1.070 (1.046–1.094)
	Q area in V6	30.252	1.002 (1.001–1.003)
	R' area in aVR	25.621	1.002 (1.001–1.003)
	Max S amplitude in V3	24.533	1.001 (1.000–1.001)
	Max S amplitude in aVR	19.300	1.001 (1.001–1.002)
	S peak time in V4	15.098	0.979 (0.969–0.990)
	R peak time in V5	11.992	0.960 (0.938–0.983)
ST‐T	Max ST level in I	62.952	0.982 (0.977–0.986)
	T area in V3	5.970	1.000 (1.000–1.000)
HCM‐apical			
P	P area in V6	14.853	1.009 (1.004–1.014)
QRS	Max S amplitude in V4	31.121	1.003 (1.002–1.004)
	S′ area in I	25.030	1.005 (1.003–1.006)
	Max S amplitude in V1	19.945	1.002 (1.001–1.002)
	QRS area in V3	18.671	1.001 (1.000–1.001)
	S peak time in V3	15.083	1.053 (1.026–1.081)
ST‐T	T peak amplitude in V4	35.975	0.998 (0.997–0.998)
	T peak time in V5	20.399	1.035 (1.020–1.050)
	ST level at J point in aVL	19.008	0.976 (0.965–0.987)
	T' area in V5	13.294	0.997 (0.996–0.999)
dHCM			
P	P area in V1	7.839	0.995 (0.992–0.999)
QRS	Q peak amplitude in V6	30.128	1.008 (1.005–1.010)
	Max R amplitude in II	25.658	0.995 (0.993–0.997)
	R peak time in III	19.008	1.040 (1.022–1.059)
	Max S amplitude in V3	9.027	1.001 (1.000–1.001)
	S peak time in V6	6.555	1.016 (1.004–1.028)
ST‐T	T' peak amplitude in I	12.584	1.022 (1.010–1.034)
	T peak amplitude in V5	10.557	0.998 (0.997–0.999)
	T duration in V3	10.140	1.020 (1.007–1.032)
	T duration in II	5.703	0.992 (0.985–0.998)

Abbreviations: dHCM, dilated phase HCM; HCM, hypertrophic cardiomyopathy; HCM‐apical, HCM with hypertrophy involving the apex; HCM‐basal, HCM with basal hypertrophy only.

**FIGURE 3:**
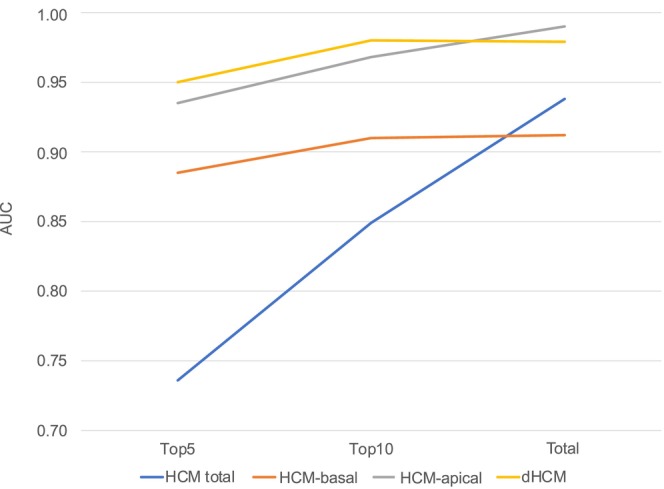
Incremental changes in AUCs of prediction models from the top 5 parameters to the top 10 parameters and total parameters for each distinct disease label (blue line: HCM total, orange line: HCM‐basal, gray line: HCM‐apex, and yellow line: dHCM). 1/*p*, reciprocal *p*‐value; AUC, area under the curve; dHCM, dilated phase hypertrophic cardiomyopathy; HCM, hypertrophic cardiomyopathy; HCM‐apical, HCM with hypertrophy involving the apex; HCM‐basal, HCM with basal hypertrophy only; P, P‐wave; QRS, QRS complex; ROC, receiver operating characteristic; ST‐T, ST‐T segment.

In Figure 3, AUCs are compared among models using the top 5 parameters, the top 10 parameters, and the total ECG parameters. For HCM‐total, the AUC increased from 0.736 (95%CI, 0.681–0.790) with the top 5 parameters to 0.849 (95%CI, 0.807–0.891) with the top 10 parameters, and 0.938 (95%CI, 0.914–0.962) with the total parameters. For HCM‐basal, the AUC increased slightly from 0.885 (95%CI, 0.847–0.922) with the top 5 parameters to 0.910 (95%CI, 0.877–0.943) with the top 10 parameters, and 0.912 (95%CI, 0.880–0.945) with the total parameters. For HCM‐apical, the AUC increased from 0.935 (95%CI, 0.894–0.976) with the top 5 parameters to 0.968 (95%CI, 0.954–0.982) with the top 10 parameters, and 0.990 (95%CI, 0.986–0.995) with the total parameters. For dHCM, the AUC increased from 0.950 (95%CI, 0.921–0.978) with the top 5 parameters to 0.980 (95%CI, 0.965–0.994) with the top 10 parameters, and 0.979 (95%CI, 0.962–0.996) with the total parameters.

### 3.5 Analysis 4: Evaluation in patients without atrial tachyarrhythmia or cardiac pacing

Out of the ECG parameters, 283 non‐collinear parameters were selected for HCM‐total, 282 for HCM‐basal, 281 for HCM‐apical, and 286 for dHCM. These were used to develop multivariate models for each disease label. ROC curves for each multivariate model are shown in Figure S3, with models developed for total ECG parameters and subsets from the P‐wave, QRS complex, or ST‐T segment.

For HCM‐total, the AUCs were 0.949 (95%CI, 0.926–0.972) for the total model, 0.774 (95%CI, 0.720–0.829) for the P‐wave model, 0.923 (95%CI, 0.893–0.954) for the QRS complex model, and 0.875 (95%CI, 0.836–0.914) for the ST‐T segment model. For HCM‐basal, the AUCs were 0.939 (95%CI, 0.914–0.964) for the total model, 0.739 (95%CI, 0.673–0.804) for the P‐wave model, 0.889 (95%CI, 0.846–0.932) for the QRS complex model, and 0.853 (95%CI, 0.800–0.907) for the ST‐T segment model. For HCM‐apical, the AUCs were 0.979 (95%CI, 0.957–1.000) for the total model, 0.837 (95%CI, 0.745–0.928) for the P‐wave model, 0.977 (95%CI, 0.964–0.990) for the QRS complex model, and 0.969 (95%CI, 0.936–1.000) for the ST‐T segment model. For dHCM, the AUCs were 0.987 (95%CI, 0.975–1.000) for the total model, 0.859 (95%CI, 0.964–1.000) for the P‐wave model, 0.942 (95%CI, 0.894–0.990) for the QRS complex model, and 0.846 (95%CI, 0.702–0.991) for the ST‐T segment model.

### 3.6 Case presentation: Change of ECG parameters in HCM patients with disease progression

In the HCM group, there are four cases with disease progression from HCM to dHCM after the enrollment. Among them, only one patient has no cardiac pacing or atrial tachyarrhythmia. The standardized values of each ECG parameter at baseline and the timing of LVEF decrease (LVEF <50%) are displayed in Figure S4.

## 4 DISCUSSION

### 4.1 Major findings

This study evaluated the diagnostic importance of 438 ECG parameters across HCM subtypes, including basal and apical forms, as well as dHCM. Our findings revealed that ST‐T segment parameters were especially significant for diagnosing HCM, with similar weight across the basal and apical subtypes. In contrast, QRS complex parameters were more pertinent for diagnosing dHCM, suggesting a shift in diagnostic emphasis as the disease progresses. Even among patients without atrial tachyarrhythmia or cardiac pacing, the diagnostic importance of QRS complex and ST‐T segment was consistent with that observed in the overall population. When comparing the diagnostic models using the top 5, top 10, and all parameters, the AUC for HCM‐total increased steadily with the number of parameters included. However, for individual HCM subtypes, the top 10 parameters demonstrated diagnostic performance comparable to that achieved with all ECG parameters.

### 4.2 Specific ECG characteristics in HCM and dHCM

Previous studies have identified abnormal ECG features in 75% to 95% of patients with HCM.^14^, ^15^, ^16^, ^22^, ^23^ However, ECG‐based detection of both HCM and dHCM remains challenging because high false‐positive rates and limited sensitivity.^24^, ^25^

Typical ECG findings of HCM include high R/S amplitude and T wave inversion in precordial leads (typically, V3‐4 or V5‐6), left atrial overload in the P‐wave, left axis deviation, and pathological Q waves. A pathological Q wave is characterized by a narrow but deep Q wave, which forms in V5 and V6 when the initial septal vector is directed from left to right, or in leads II, III, and aVF when the vector is oriented upward. In our study, the top 10 parameters for predicting HCM total included P areas in I, II, and aVL. Typically, high‐amplitude P waves in leads II, III, and aVF indicate right atrial overload, while a biphasic P wave in lead II or an increase in the terminal negative component of the P wave in lead V1 suggests left atrial overload. Interestingly, our top 10 parameters for HCM total included not only the P area in II but also the P areas in I and aVL. Two reports suggest that the amplitude of the P wave in lead I, rather than in lead II, is associated with the progression of electrical remodeling in the left atrium,^26^, ^27^ where the direction toward the left free wall may represent the interatrial conduction time and increase the risk of atrial fibrillation.^26^, ^27^, ^28^ In line with this, P areas in I and aVL may represent atrial remodeling related to LVH. Our top 10 parameters for HCM total also included both Q areas in III and V6, which correspond to the initial septal vector towards the upper direction and the left to right direction, respectively. The R axis, QRS area in V3, and Max R amplitude in II can be interpreted as the direct reflection of LVH. Interestingly, Max S amplitude in V4 and S area in V5 were strongly associated with HCM total. Commonly, the voltage of the S wave in V1 combined with the R wave in V5 or V6 is used to diagnose LVH, according to the Sokolow –Lyon criteria^29^ However, changes with a short R and deep S in V4 to V6 are observed with the increase of septal LVH, which appears from V4 to V6 in a stepwise manner.^30^

The top 10 parameters for predicting HCM basal can be similarly interpreted as those for HCM total, at least in part, and include high R/S amplitude and T wave inversion in precordial leads (R peak time in V4, R peak time in V4 and V5, and T area in V3, and Max ST level in I), left atrial overload in P‐wave (P area in I), and pathological Q wave (Q area in V6). The S wave (S peak time in V4) can also be similarly interpreted as for HCM total. However, it is difficult to understand the relationship of R' area and Max S amplitude in aVR with HCM basal. As aVR lead can serve as a reciprocal to leads I or II, it may respond to the S wave or R wave in these leads.

The top 10 parameters for predicting HCM apical included T peak amplitude in V4 (negative association), T peak time in V5 (positive association), and T' area in V5 (negative association), which would respond to the giant negative T wave. Other parameters were interpretable in line with the parameters for HCM total, including high R/S amplitude and T wave inversion in precordial leads (QRS area in V3, ST level at J point in aVL, and the aforementioned T parameters), and left atrial overload in the P‐wave (P area in V6). Parameters that responded to pathological Q waves were not included in the top 10 parameters for HCM apical, consistent with previous reports that pathological Q waves are not considered a characteristic ECG feature for HCM apical,^31^ which may distinguish it from other forms of HCM.^32^ S wave (Max S amplitude in V1 and V4, and S peak time in V3) can be similarly interpreted as for HCM total but seems to be more prominently observed in HCM apical compared with HCM basal.

Among the top 10 parameters for predicting dHCM, the top 3 parameters were QRS parameters, including Q peak amplitude in V6, Max R amplitude in II, and R peak time in III, which differ from the characteristics in other types. To our knowledge, only one study, published in 1999, has analyzed dHCM‐specific ECG features, identifying a progressive decrease in S wave amplitude in V1 combined with the R wave in V5 over 20 years as correlating with ventricular tachycardia or poor prognosis.^33^ This ECG parameter change reported by Doi et al. reflected reduced LVH compared with HCM in the early phase. Although these parameters are not included in our top 10, it may be natural to exclude them, as they reflect differences from HCM rather than the overall picture of dHCM. Our top 10 parameters for predicting dHCM included high R/S amplitude and T wave inversion in both limb and precordial leads (Max R amplitude in II, R peak time in III, T' peak amplitude in I, T duration in II and V3, and T peak amplitude in V5), left atrial overload in the P‐wave (P area in V1), and pathological Q waves (Q peak amplitude in V6). The pathological Q wave in dHCM is thought to reflect the cardiac remodeling rather than reflecting LVH. ECG features predicting heart failure with reduced ejection fraction have been reported to include prolonged QRS duration, time to intrinsicoid deflection, prolonged QT interval, abnormal QRS‐T axis, LVH, and ST‐T wave abnormalities,^34^ which partially coincide with our top 10 parameters for dHCM. Thus, the ECG findings of dHCM are characterized by mild hypertrophy, cardiac enlargement, and reduced left ventricular systolic function, and compared with HCM, they consist of a combination of non‐specific findings.

### 4.3 Analysis of ECG parameter importance for HCM and dHCM

Recent AI‐based ECG models have achieved high diagnostic performance, with AUC values around 0.90 for both HCM and dHCM.^18^, ^19^, ^20^, ^35^ However, clinical translation of these insights requires a clear understanding of the specific ECG parameters responsible for the diagnostic process. While AI models can indicate regions of interest, interpreting individual ECG features continues to rely on clinical expertise.

In this study, we used traditional statistical methods to assess the significance of individual ECG parameters in diagnosing HCM subtypes. For both basal and apical HCM, the ST‐T segment parameters demonstrated the highest diagnostic importance, with about half surpassing a 1/ *P*‐value of 10^4^. The QRS complex also showed considerable importance, with one‐third of its parameters exceeding this threshold, while the P‐wave segment had limited predictive value, with only 10% of its parameters reaching the 10^4^ threshold. These findings align with segment‐specific AUCs, which were highest for the ST‐T segment, followed by the QRS complex and P‐wave.

In the multivariate model, the top 10 parameters for HCM‐basal included two ST‐T segment parameters, seven QRS complex parameters, and one P‐wave parameter, achieving predictive performance similar to that of the total parameters. Similarly, for HCM‐apical, the top 10 parameters included four from the ST‐T segment, five from the QRS complex, and one from the P‐wave, yielding comparable predictive performance.

For dHCM, the QRS complex showed the highest parameter importance, with one‐fourth of its parameters exceeding the 10^4^ threshold, followed by the ST‐T segment, with one‐sixth meeting this criterion. These proportions were lower than those observed in HCM‐basal and HCM‐apical. The P‐wave segment's predictive value remained limited, with only 10% of parameters exceeding the 10^4^ threshold, similar to HCM‐basal and HCM‐apical. Segment‐specific AUCs for dHCM were highest for the QRS complex, followed by the ST‐T segment and P‐wave. Although individual parameter importance for dHCM was relatively low, segment‐specific AUCs were high. The top 10 parameters for dHCM included five from the QRS complex, four from the ST‐T segment, and one from the P‐wave, again providing predictive performance comparable to the total parameter set.

These findings highlight two main points. First, the shift in importance from ST‐T segment to QRS complex parameters as HCM progresses to dHCM may aid in the early detection of functional deterioration. Second, although the predictive performance of the model using the top 10 parameters was lower than that of the total parameter set for HCM total, in each HCM subtype, the top 10 parameters achieved predictive performance comparable to the total parameter set. This suggests that considering tailoring AI models to specific disease subtypes could enhance efficiency. In our previous work on AI‐enhanced ECG for predicting HCM, the AUC for HCM total was 0.854 with a basic CNN model, which improved to 0.887 after integrating diagnostic probabilities from modes specific to HCM subtypes.^19^

### 4.4 Limitations

This study has several limitations. First, it was conducted at a single cardiovascular hospital in an urban area, which may limit the generalizability of the findings. Second, although HCM diagnoses were confirmed through cardiac imaging, clinical history, and family history, the absence of genetic testing may have resulted in the inclusion of cases with conditions mimicking HCM, such as hypertensive hypertrophy. Third, the relatively small sample size of HCM and dHCM cases limited the statistical power. Fourth, we applied a stringent threshold of *p* = .0001 (1/*p* = 10^4^) for HPI, which is more conservative than the conventional *p* = .05, aiming to emphasize strong statistical significance. However, this threshold does not reflect effect sizes, which warrant caution in interpretation.^36^ To address this, we evaluated AUCs for multivariate models with integrated parameters, including ECG segment‐specific parameter sets or the top 5 and top 10 parameters.

## 5 CONCLUSIONS

As HCM progresses from basal or apical types to dHCM, a shift in HPI from the ST‐T segment to the QRS complex was observed, offering clinically valuable insights. For HCM subtypes, the predictive performance of the top 10 ECG parameters was comparable to that of the full parameter set, suggesting an efficient approach to developing AI‐based diagnostic models.

## AUTHOR CONTRIBUTIONS

All authors take responsibility for all aspects of the reliability and freedom from bias of the data presented and their discussed interpretation.

## FUNDING INFORMATION

This research did not receive any specific grants from funding agencies in the public, commercial, or not‐for‐profit sectors.

## CONFLICT OF INTEREST STATEMENT

Dr. Suzuki received lecture fees from Daiichi Sankyo and Bristol‐Myers Squibb. Dr. Yamashita received research funds and/or lecture fees from Daiichi Sankyo, Bayer Yakuhin, Bristol‐Myers Squibb, Pfizer, Nippon Boehringer Ingelheim, Eisai, Mitsubishi Tanabe Pharm, Ono Pharmaceutical, and Toa Eiyo.

## ETHICS STATEMENT

This study was performed in accordance with the Declaration of Helsinki (revised in 2013) and the Ethical Guidelines for Medical and Health Research Involving Human Subjects (Public Notice of the Ministry of Education, Culture, Sports, Science and Technology, and the Ministry of Health, Labor and Welfare, Japan, issued in 2017). Written informed consent was obtained from all participants. The study protocol was reviewed by the Institutional Review Board of the Cardiovascular Institute.

## APPROVAL OF THE RESEARCH PROTOCOL

Not applicable.

## ANIMAL STUDIES

Not applicable.

## CONSENT FOR PUBLICATION

Not applicable.

## INFORMED CONSENT

Written informed consent was obtained from all participants.

## PERMISSION TO REPRODUCE MATERIAL FROM OTHER SOURCES

None.

## REGISTRY AND THE REGISTRATION NUMBER

Not applicable.

## Supporting information


Figure S1.

## Data Availability

Data cannot be shared publicly because of a lack of such a description in the study protocol and informed consent. Data are available from the Ethics Review Committee at the Cardiovascular Institute for researchers who meet the criteria for access to confidential data (contact via the corresponding author).
