# Supplementary material for: Electrocardiographic parameter profiles for differentiating hypertrophic cardiomyopathy stages
Source: J Arrhythm. 2025 Mar 4;41(2):e70031. doi: 10.1002/joa3.70031 (PMC11876995; doi:10.1002/joa3.70031)

Supplementary Figure 1. ECG parameters

Parameters used for analysis: 438 parameters

(1) Lead-nonspecific parameters (N = 6):

P-R Interval, P axis, QRS Duration, QTc Calculation (QTc Bazett), R axis, T axis

(2) Lead-specific parameters (N = 432 [36 x 12 leads]):

ST at J Point, P Area, P‘ Area, P Area (Full), P Peak Time, P’ Peak Time, P Peak Amplitude, P' Peak Amplitude, P Duration, P' Duration, QRS Area, Q Area, Q Peak Amplitude, Q Duration, R Area, R' Area, R Peak Time, R Duration, R' Duration, S Area, S' Area, S Peak Time, S Duration, S' Duration, T Area, T' Area, T Area (Full), T Peak Time, T Peak Amplitude, T' Peak Amplitude, T Duration, T' Duration, Minimum ST level, Max R Amplitude, Maximum ST level, Max S Amplitude

Parameters excluded: 201 parameters

(1) Lead-nonspecific parameters (N = 9):

P Onset, P Offset, QRS Count, QTc Framingham, QTc Fridercia, Q-T Interval, Q Onset, Q Offset, T Offset

(2) Lead-specific parameters (N = 192 [16 x 12 leads]):

P Onset Amplitude, QRS Balance, QRS Deflection, QRS Intrinsicoid, Q Peak Time, R‘ Peak Time, R Peak Amplitude, R' Peak Amplitude, S' Peak Time, S Peak Amplitude, S' Peak Amplitude, T' Peak Time, T End, ST at End ST, ST at Mid ST, Special T

Supplementary Figure 2. Typical ECG

A. HCM-total

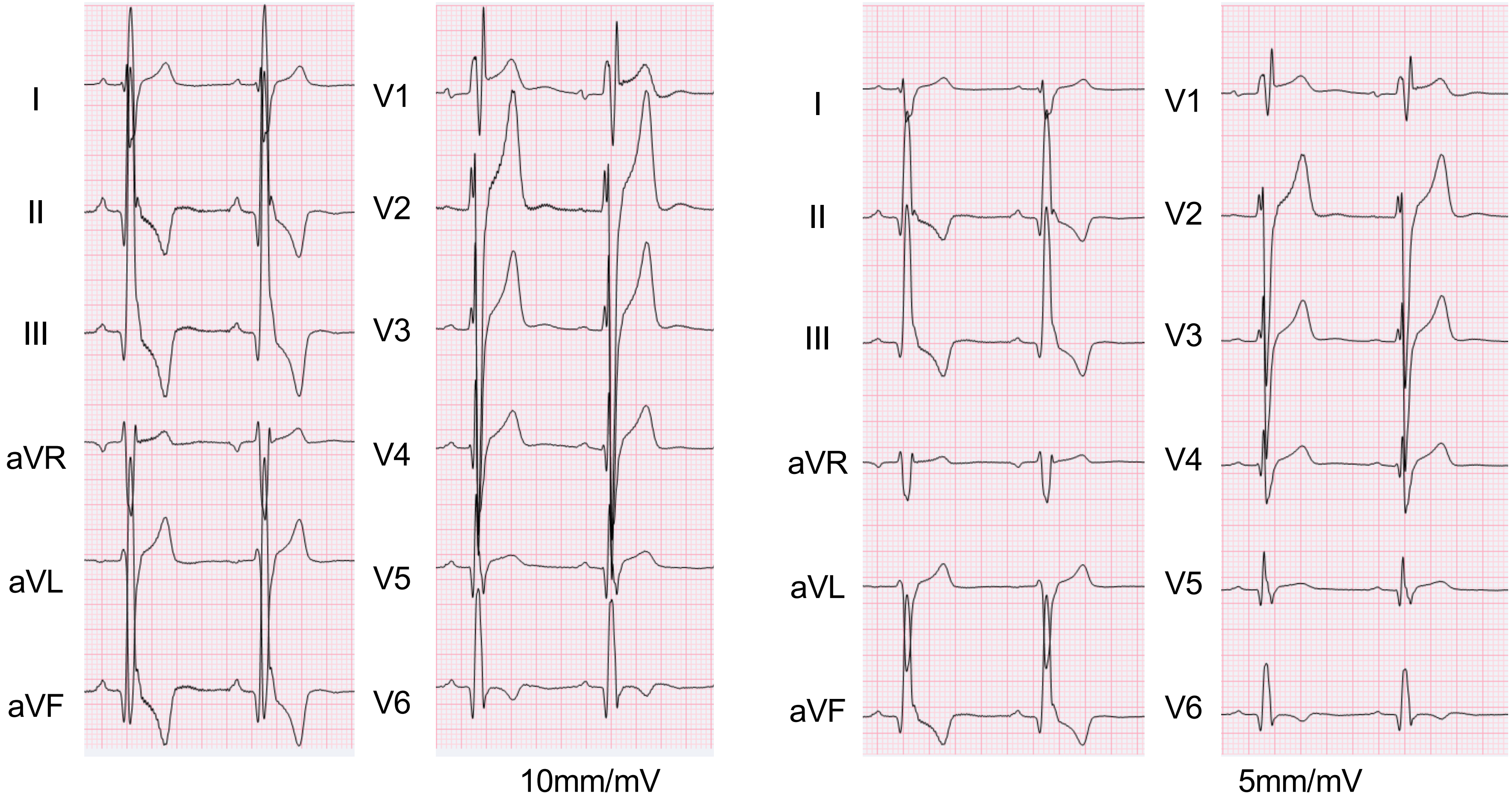

Supplementary Figure 2. Typical ECG

B. HCM-basal

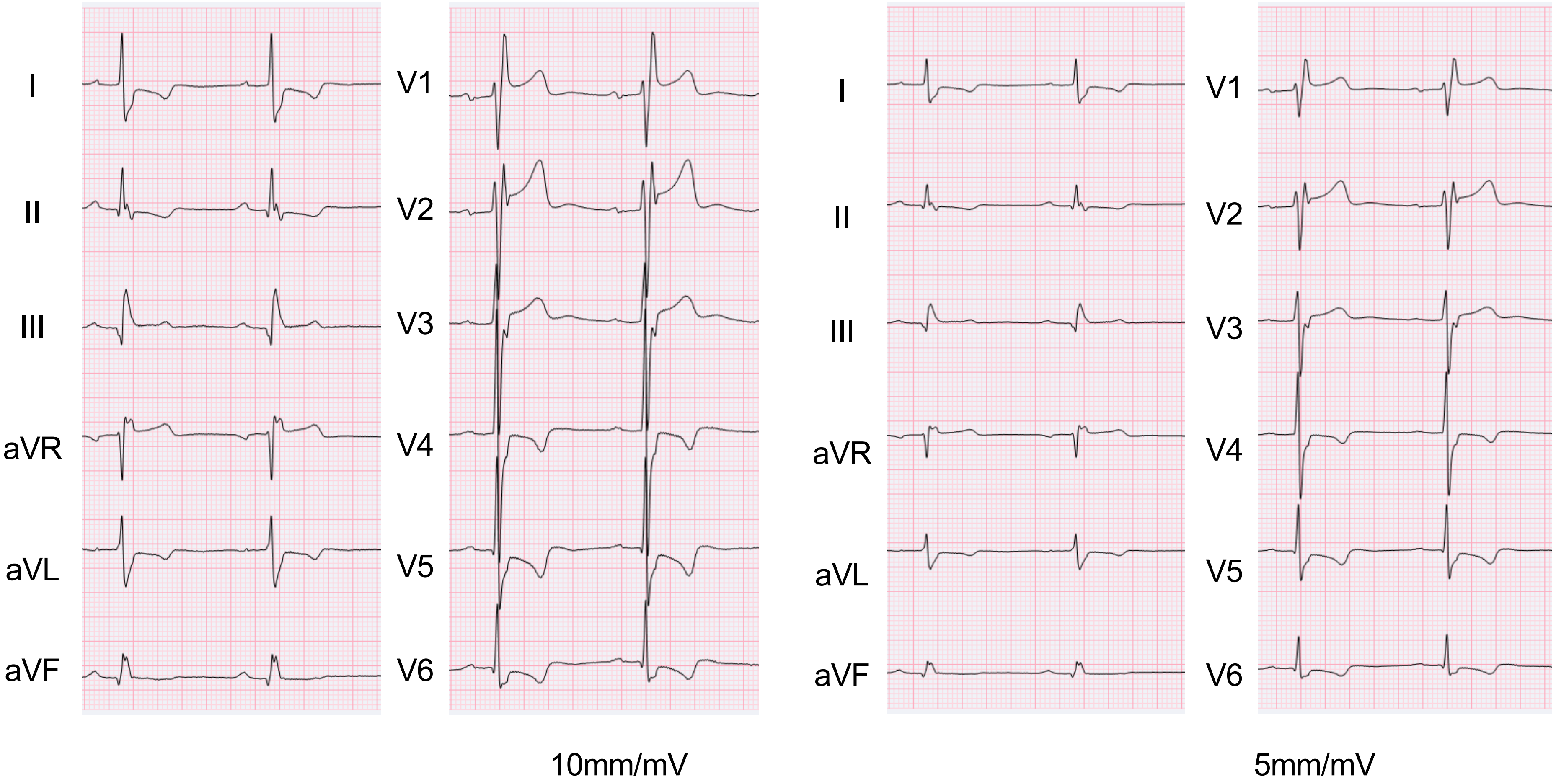

Supplementary Figure 2. Typical ECG

C. HCM-apical

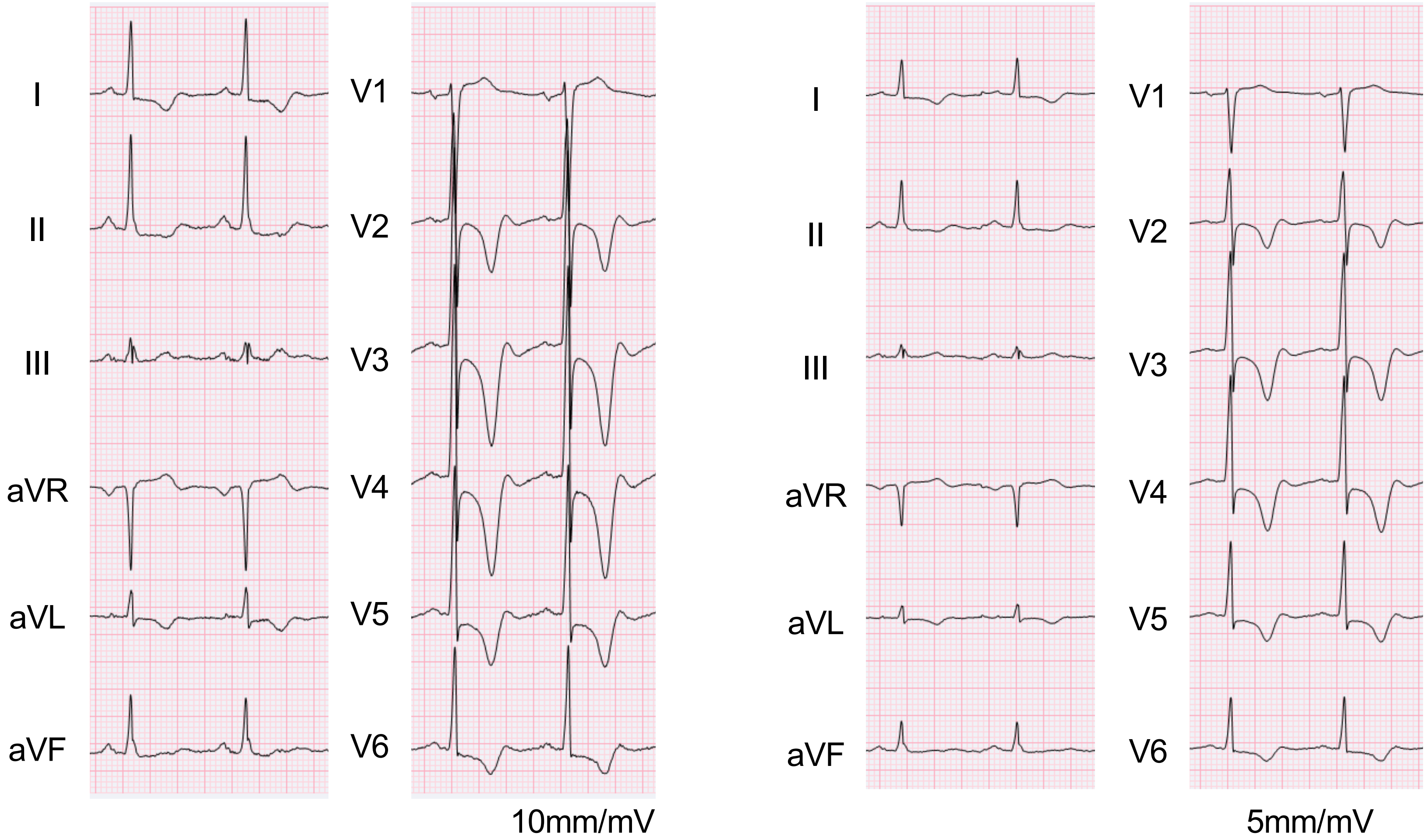

Supplementary Figure 2. Typical ECG

D. dHCM

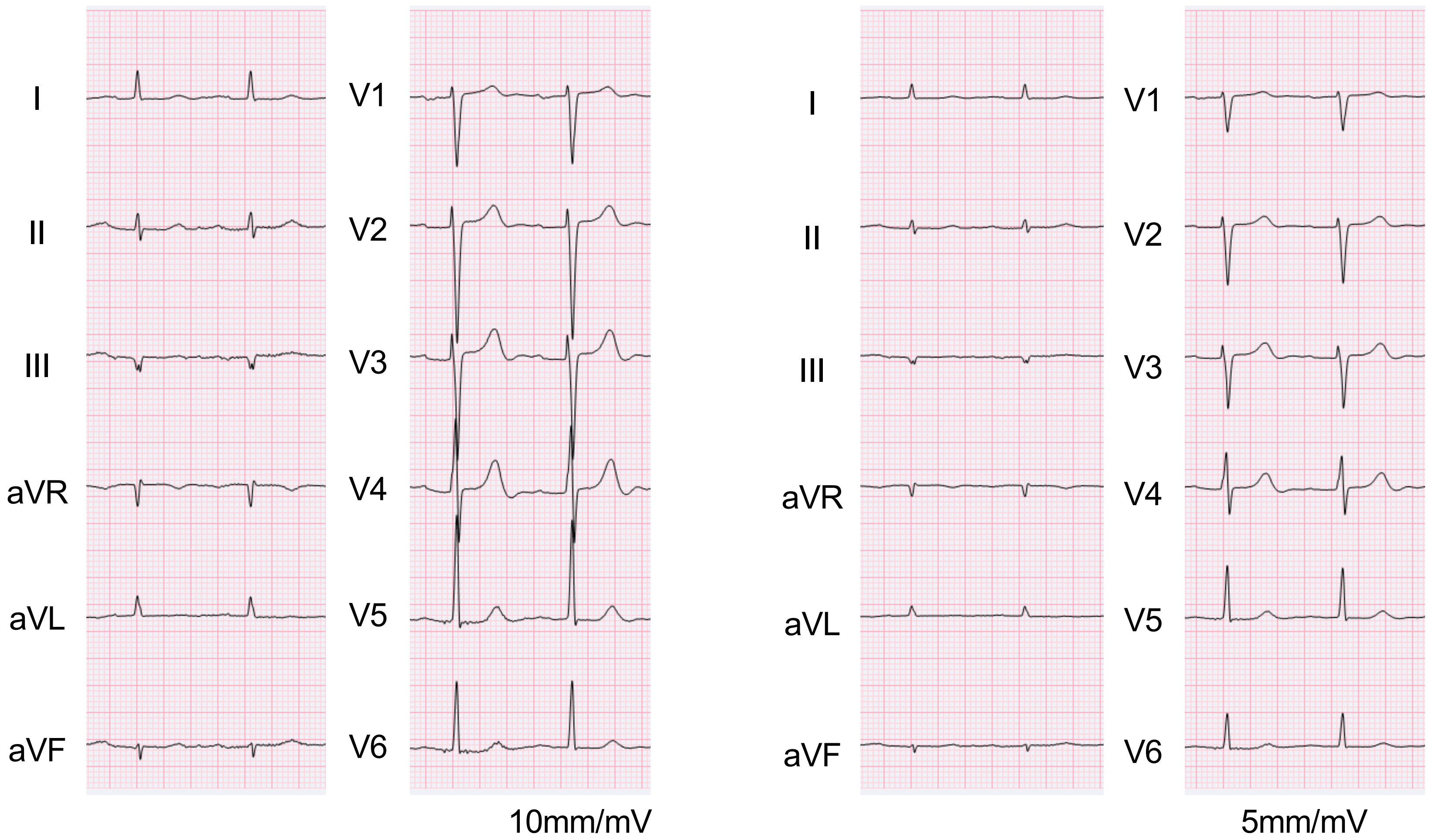

Supplementary Figure 3. ROC curve for each distinct labels using all parameters, parameters for P-wave, QRS complex, and ST-T segment in patients without atrial tachyarrhythmia or cardiac pacing

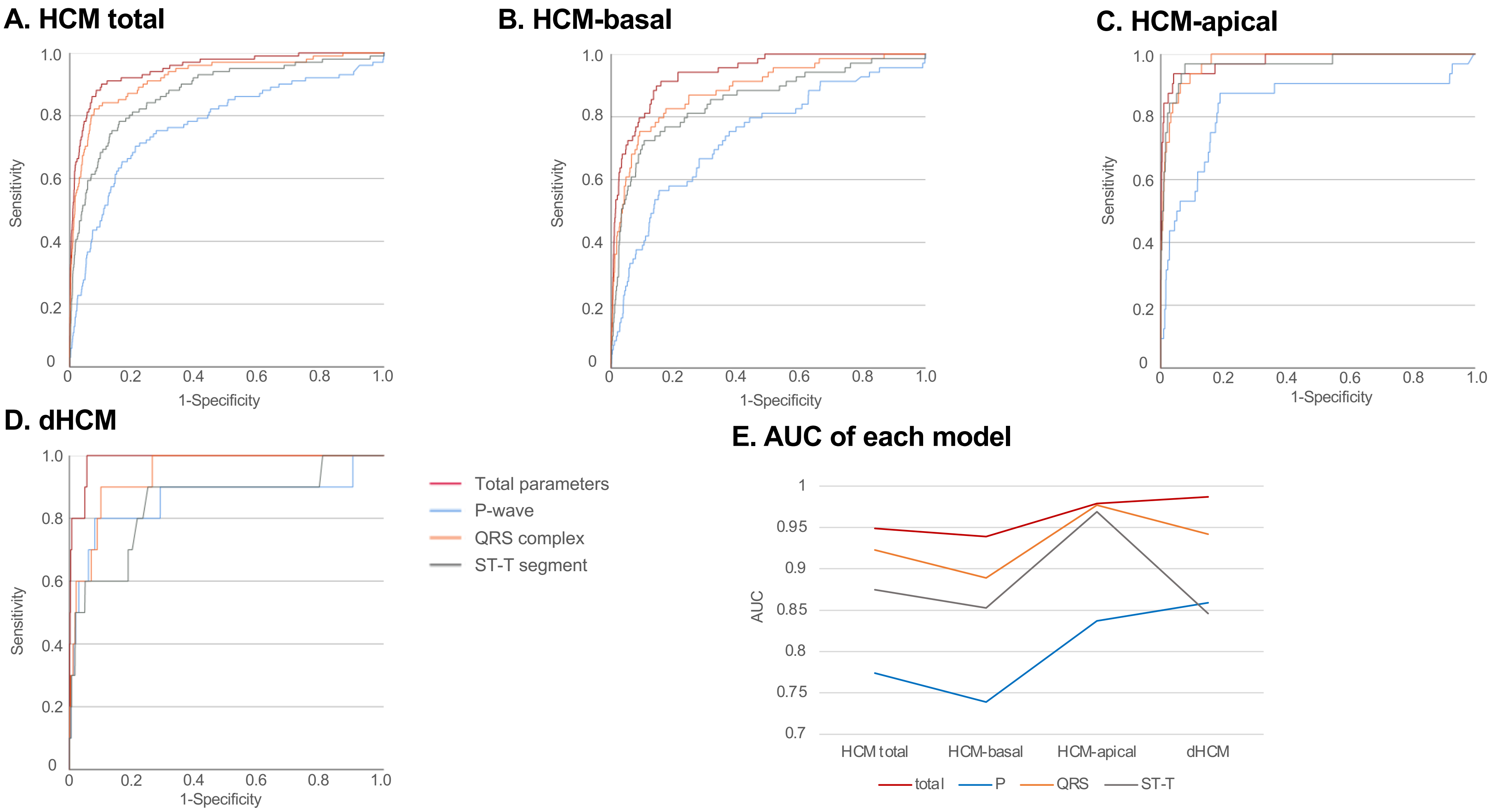

Supplementary Figure 4. The standardized values of each ECG parameter in a patient with disease progression from HCM to dHCM at baseline and at the time of LVEF decline (LVEF < 50%).

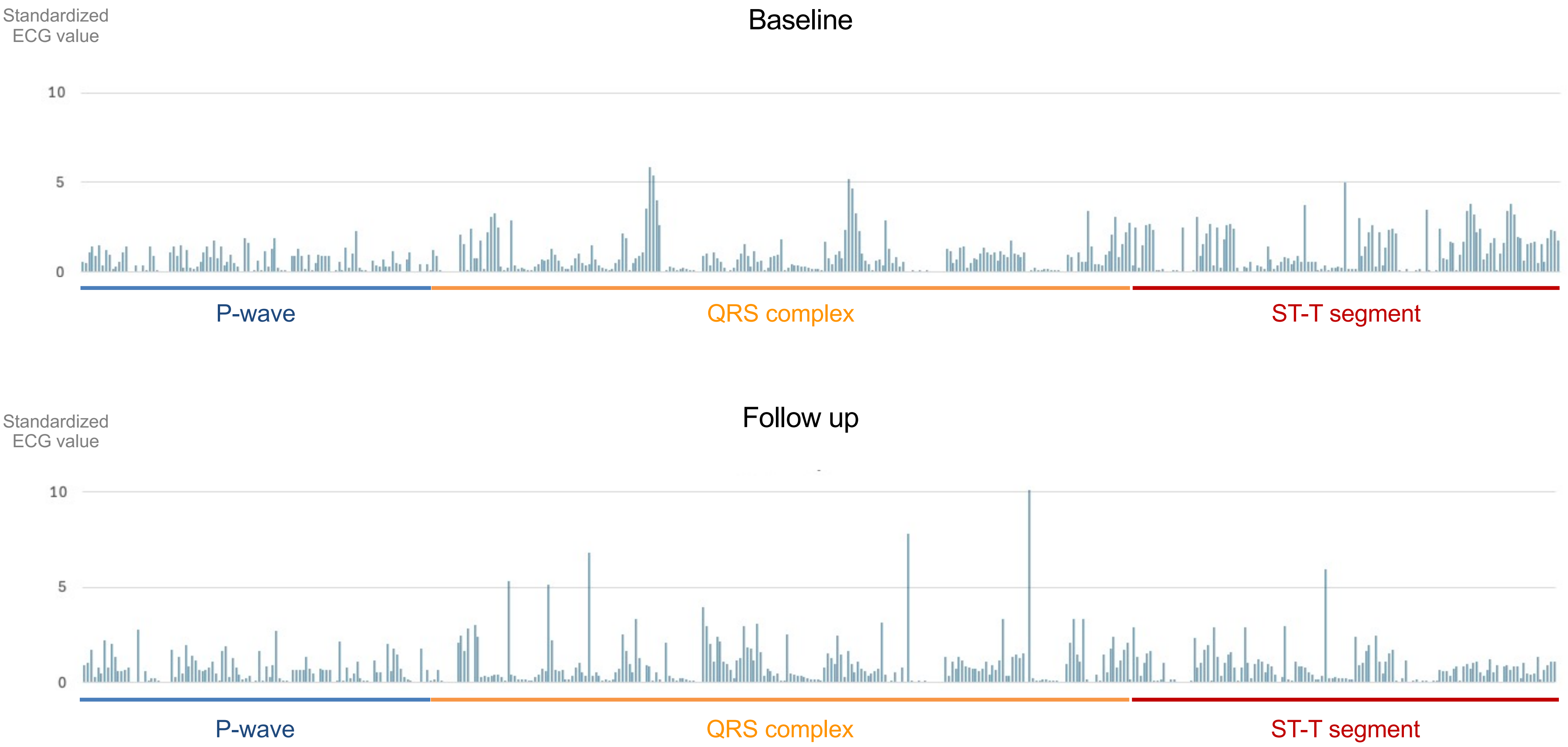

Supplement: Supplementary file 1 — Figure S1. [file JOA3-41-e70031-s001.pdf]
